# Unexpected absence of ribosomal protein genes from metagenome-assembled genomes

**DOI:** 10.1038/s43705-022-00204-6

**Published:** 2022-11-28

**Authors:** Kazumori Mise, Wataru Iwasaki

**Affiliations:** 1 Department of Biological Sciences, Graduate School of Science, The University of Tokyo. Bunkyo-ku, Tokyo, 113-0032 Japan; 2 National Institute of Advanced Industrial Science and Technology, Sapporo, Hokkaido, 062-8517 Japan; 3 Department of Integrated Biosciences, Graduate School of Frontier Sciences, The University of Tokyo, Kashiwa, Chiba, 277-0882 Japan; 4 Department of Computational Biology and Medical Sciences, Graduate School of Frontier Sciences, The University of Tokyo, Kashiwa, Chiba, 277-0882 Japan; 5 Atmosphere and Ocean Research Institute, The University of Tokyo, Kashiwa, Chiba, 277-0882 Japan; 6 Institute for Quantitative Biosciences, The University of Tokyo, Bunkyo, Tokyo, 113-0032 Japan; 7 Collaborative Research Institute for Innovative Microbiology, The University of Tokyo, Bunkyo, Tokyo, 113-0032 Japan

**Keywords:** Metagenomics, Environmental microbiology

## Abstract

Metagenome-assembled genomes (MAGs) have revealed the hidden diversity and functions of uncultivated microbes, but their reconstruction from metagenomes remains a computationally difficult task. Repetitive or exogenous sequences, such as ribosomal RNA and horizontally transferred genes, are frequently absent from MAGs because of misassembly and binning errors. Here, we report that ribosomal protein genes are also often absent from MAGs, although they are neither repetitive nor exogenous. Comprehensive analyses of more than 190,000 MAGs revealed that these genes could be missing in more than 20–40% of near-complete (i.e., with completeness of 90% or higher) MAGs. While some uncultivated environmental microbes intrinsically lack some ribosomal protein genes, we found that this unexpected absence is largely due to special evolutionary patterns of codon usage bias in ribosomal protein genes and algorithmic characteristics of metagenomic binning, which is dependent on tetranucleotide frequencies of contigs. This problem reflects the microbial life-history strategy. Fast-growing microbes tend to have this difficulty, likely because of strong evolutionary pressures on ribosomal protein genes toward the efficient assembly of ribosomes. Our observations caution those who study genomics and phylogeny of uncultivated microbes, the diversity and evolution of microbial genes in the central dogma, and bioinformatics in metagenomics.

## Introduction

Reconstructing metagenome-assembled genomes (MAGs), also known as genome-resolved metagenomics, has significantly expanded our knowledge of microbial diversity and function. Notable examples include the discovery of major phylogenetic groups without precedent isolates, namely Candidate Phyla Radiation bacteria, DPANN archaea [1–3], and crAssphage [4], as well as the identification of comammox bacteria [5, 6]. Furthermore, mining public metagenomic databases has generated myriads of MAGs and expanded our knowledge of the diversity and potential functions of environmental microbes [7, 8].

There are many bioinformatic tools that enable fast and memory-efficient reconstruction of MAGs [9–13]. Reconstructing MAGs involves two main processes: (1) assembling short and fragmented reads into long contigs and (2) binning contigs from the same species. Both processes are computationally difficult, and the accuracy of MAGs has been repeatedly discussed [14]. First, misassemblies frequently occur in repetitive sequences, such as ribosomal RNA genes. Many prokaryotes harbor multiple copies of ribosomal RNA operons with highly similar nucleotide sequences, which can be erroneously merged during the assembly process [15]. Long repeat sequences also produce similar problems and are difficult to assemble [14]. Second, binning errors frequently arise in exogenous sequences, such as plasmids and genomic islands [16]. One straightforward approach to cluster contigs is to put together contigs with similar sequencing depths, but this cannot distinguish microbes existing in similar amounts. Therefore, popular binning tools, such as MetaBAT [11], MaxBin [10], and CONCOCT [17], use tetranucleotide frequencies to cluster contigs, including those without marker genes, in addition to sequence depths information. This strategy is based on the empirical knowledge that a tetranucleotide frequency is a suitable fingerprint representing prokaryotic phylogeny [18]. However, plasmids and genomic islands are transferred from one cell to another and bear little hallmark of the host prokaryotes.

Here, we report another unnoticed hotspot of systematic errors in MAG reconstruction: ribosomal protein genes. Comprehensive analyses of tens of thousands of MAGs, as well as those of single-cell amplified genomes (SAGs) and unassembled metagenomic sequences, have revealed that ribosomal protein genes are frequently absent from MAGs, although they are neither repetitive nor exogenous. We also found that distinct tetranucleotide frequencies around ribosomal protein genes caused frequent losses during the binning process, and bacterial life-history strategies affected this tendency.

## Methods

### MAG and SAG datasets

We used six datasets of MAGs reported in previous studies, encompassing various types of environments (Table 1). Four datasets consisted of MAGs reconstructed from Illumina short-read sequencing of diverse environments, namely seawater [7], the human gut [8], the chicken gut [19], and the rice phyllosphere [20]. We obtained another dataset from the Genomic catalog of Earth’s Microbiomes (GEM) [21]. In contrast to the above four datasets, the GEM contains MAGs from various types of sequencers, including long- and short-read sequencers. To compare MAGs reconstructed with and without long-read sequencers, we also used a catalog of wastewater MAGs reconstructed by hybrid assembly of long-read (Oxford Nanopore) and short-read (Illumina NextSeq) sequences [22].

**Table 1:** Description of six MAG datasets used in this study.

Dataset name	Number of genomes^a^	Taxonomic annotation	Assembly	Binning	Quality check	Reference
Seawater MAGs	Short-read: 47,120	GTDB-Tk v1.3.0	MEGAHIT v1.1.4	MaxBin v2.2.6 MetaBAT v2.12.1 CONCOCT v1.0.0 Ensembled using MetaWRAP v1.2.1	CheckM v1.0.13	[7]
Human gut MAGs	Short-read: 90,301	GTDB-Tk v2.1.0^b^	MEGAHIT v1.1.3	MaxBin v2.2.4 MetaBAT v2.12.1 MetaBAT1 Ensembled using MetaWRAP v1.0	CheckM v1.0.7	[8]
Chicken gut MAGs	Short-read: 12,232	GTDB-Tk; version not disclosed	MEGAHIT v1.1.3	MaxBin v2.2.6 MetaBAT v2.12.1 CONCOCT v1.1.0 Ensembled using MetaWRAP v1.2.1	CheckM v1.0.12	[19]
Rice phyllosphere MAGs	Short-read: 503	GTDB-Tk v1.4.1	MEGAHIT; version not disclosed	MaxBin v2.2.7 MetaBAT v2.12.1 Vamb v3.0.2 Ensembled using DASTool v1.1.1	CheckM v1.0.13	[20]
Genomic catalog of Earth’s Microbiomes (GEM)	Short-read: 45,695 Long-read or hybrid: 369	GTDB-Tk; version not disclosed	Not standardized	MetaBAT v0.32.4/5	CheckM v1.0.11	[21]
Wastewater long-read MAGs	Hybrid: 1080	GTDB-Tk v1.0.2	CANU v1.8	MetaBAT v2.12.1 MaxBin v2.2.7 Ensembled using DASTool v1.1.1	CheckM v1.0.11	[22]

^a^Number of bacterial MAGs.

^b^Annotated in this study.

We referred to the JGI Genome Online Database [23], NCBI Sequence Read Archive [24], and supplemental tables attached to the abovementioned literature to identify the types of sequencing platforms used for reconstructing each MAG. Some MAGs in the GEM lacked available records of sequencing platforms or were reconstructed from Sanger sequencing, and they were excluded from this study. The remaining MAGs were classified into two groups: ones reconstructed from short-read sequences only and ones reconstructed using long-read sequences (i.e., hybrid assembly or long-read assembly).

Each of the six datasets was accompanied by completeness and contamination scores calculated using CheckM v1.0.7–13 [25] and taxonomic assignments based on the Genome Taxonomy Database (GTDB) [26] except for the human gut dataset. MAGs from the human gut dataset were taxonomically annotated using GTDB-Tk v2.1.0 [27–32]. Only bacterial genomes were analyzed in this study. Genomes annotated as archaeal or without valid domain-level annotations (according to GTDB-Tk) were excluded. Table 1 summarizes the number of genomes, versions of CheckM and GTDB-Tk, and the assembly and binning methods.

To compare the MAGs with genomes free of metagenomic binning, we also analyzed SAGs from seawater (*n* = 12,715) and mouse gut (*n* = 31) samples [33, 34]. Five SAGs from seawater were unavailable in GenBank and were not used in this study. Qualities of the SAGs were assessed using CheckM v1.1.3 [25] (“lineage_wf” command and default parameters), and those with completeness scores of >90% were used.

Protein-coding sequences (CDSs) on the MAGs and SAGs were predicted using Prodigal v2.6.3 [35] and subsequently annotated using KofamScan v1.3.0 [36] with KOfam profiles version 2021-04-01 (the same applies below). FASTQ and FASTA files were formatted using SeqKit [37] where needed.

### Core ribosomal protein and single-copy gene datasets

We downloaded 3315 bacterial genomes as the genomes of mostly pure isolates from RefSeq [38] as of April 8, 2022. We randomly sampled one genome from each genus and discarded any genome without solid annotations at the genus level. CDSs on these genomes were predicted and annotated using Prodigal and KofamScan, respectively. From these 3315 genomes, we randomly sampled one genome per class (93 genomes). We identified 42 core ribosomal protein genes and 32 other single-copy genes (Supplementary Table S1), each of which existed exactly one copy in >95% of the 93 genomes.

### Shotgun metagenome analysis

To examine if MAGs lacking some ribosomal protein genes are artifacts in the bioinformatic processes or not, we compared frequencies of ribosomal protein genes in MAGs and (pre-assembly) raw reads from shotgun metagenomics. We downloaded 11 shotgun metagenomic datasets from agricultural soil, seawater, and the human gut [39–41] (Supplementary Table S2). All data were generated by short-read Illumina sequencing. Paired-end reads were merged using the “fastq_mergepairs” command of USEARCH v11.0.667 [42] for the soil and seawater metagenomes, with the options “-fastq_maxdiffs 5 -fastq_minovlen 15 -fastq_allowmergestagger.” The inserted library size of the human gut metagenomes was too long for the paired-end reads to be merged, and read1 was used for further analysis. Sequences with expected errors of >0.5 bases were removed using the “fastq_filter” command in USEARCH.

The processed reads were functionally annotated using DIAMOND v2.0.14 [43] and the Kyoto Encyclopedia of Genes and Genomes (KEGG) database (obtained from the KEGG FTP service, as of April 10, 2022) [44]. We performed a two-step homology search [45] to reduce computational cost. First, we mapped all query sequences onto a small database consisting of core gene sequences (options: “blastx --sensitive -e 1e-5”). Queries with any hit in the first step (translated sequences) were then subjected to a homology search against the full KEGG database (options: “blastp --sensitive -e 1e-10 -k 200”). We obtained 200 hits and extracted the top hit among them for downstream analysis to mitigate the inaccuracy induced by DIAMOND heuristics. The number of reads encoding each KEGG ortholog group was converted to reads per kilobase per million reads (RPKM) to normalize the effect of each KEGG ortholog group’s length. The average length of sequences belonging to each KEGG ortholog group was used to calculate the RPKM. The Brunner–Munzel test was used to test the null hypothesis that RPKMs of ribosomal protein genes are not significantly different from those of the other single-copy genes.

Next, using those metagenomic sequences, we examined if ribosomal protein genes are often absent in MAGs because those MAGs are insufficiently assembled or not. Three subsets of reads with different depths (2 × 10^6^ reads, 4 × 10^6^ reads, and 1 × 10^7^ reads) were generated from each sample, amounting to 33 datasets. Each dataset was independently assembled using two assemblers (MEGAHIT v1.2.9 [9] and metaSPAdes v3.15.4 [13]), producing 66 sets of contigs. MEGAHIT was run with the options “--k-min 41 --k-max 121 --k-step 10”, whereas metaSPAdes was run with the default parameters under the “--meta” flag. The CDSs on the contigs were predicted and functionally annotated using Prodigal and KofamScan, respectively. Contigs with one or more ribosomal protein gene were selected, and their N50, N90, and median lengths were calculated using SeqKit v2.2.0. Similarly, contigs bearing a CDS with any K number assignment were selected, and their N50, N90, and median lengths were determined.

### Tetranucleotide frequency analysis

To investigate if tetranucleotide frequency biases around ribosomal protein genes can lead to binning errors and frequent losses of ribosomal protein genes from MAGs, we used the 3315 RefSeq genomes (one genome per genus). For each genome, the contigs were randomly sorted, concatenated in tandem, and split into multiple subsequences (25,000–26,000 bases each, based on the N50 statistics of short-read MAGs (Table 1, corresponding to the 57.1–58.1 percentile). We obtained 7–526 subsequences (mean: 157.1 subsequences) per genome. Tetranucleotide frequencies were calculated for each subsequence and its reverse complement. Here we considered the fact that the frequencies of 4^4^ (=256) patterns of tetranucleotides are not independent of each other. For example, the frequencies of reverse complement sequences (e.g., ATTC and GAAT) are exactly the same. In addition, for example, the frequencies of CAAA and AAAT are interdependent with each other, because both of them must correlate with the frequency of AAA. To eliminate such redundancy, we used the frequencies of 103 tetranucleotides (Supplementary Table S3) that can be regarded as independent [46].

CDSs on these subsequences were predicted and functionally annotated using Prodigal and KofamScan, respectively. For each genome, a weighted network of the subsequences was constructed, where the vertices are the subsequences and the weight of each edge is 1–*d*, where *d* is the Euclidean distance between their tetranucleotide frequencies. It should be noted that MetaBAT and MaxBin also used the Euclidean distance between tetranucleotide frequencies [10, 11]. We calculated the degree centrality (i.e., the sum of all the edge weights) of each vertex as a metric of the typicalness of that subsequence (i.e., whether that subsequence presented typical or atypical tetranucleotide frequencies within the genome). We calculated the percentile ranks of the degree centralities of subsequences that coded ribosomal protein genes (Supplementary Table S1), clustered regularly interspaced short palindromic repeat (CRISPR)-related genes (KEGG orthologs with annotations containing the word “CRISPR” or “CRISP”), transposon-related genes (KEGG orthologs with annotations containing the word “transpos*”), and glycolysis genes (KEGG orthologs marked as such in KEGG Pathway) among all the subsequences. Only orthologous groups present in >10% of the 3 315 genomes were used.

### Analysis of ribosomal protein gene distributions in bacterial genomes

We downloaded five phylogenetically distant and complete bacterial genomes (i.e., genomes assembled into single contigs) from the KEGG database (T00007 [*Escherichia coli* str. K-12 substr. MG1655], T00010 [*Bacillus subtilis* subsp. subtilis 168], T00015 [*Mycobacterium tuberculosis* str. H37Rv], T00035 [*Pseudomonas aeruginosa* sp. PAO1], and T00109 [*Bradyrhizobium diazoefficiens* str. USDA 110]), and visualized the distributions of ribosomal protein genes on them. The positions of ribosomal protein genes on each genome were retrieved using the KEGG FTP service.

We also examined the hypothesis that ribosomal protein genes encoded in long operons are more likely to be absent from MAGs. Ribosomal protein genes whose start-codon positions are within 1000 bases of those of the adjacent genes were regarded to be on the same operon. For each ribosomal protein gene, an average operon length on the five genomes was calculated. Those lengths were compared with proportions of MAGs harboring those genes for each of the five MAG datasets (short-read MAGs only).

### Analysis of CRISPR and transposase genes

We then compared the frequencies of the losses of ribosomal protein genes and the amount of other known hotspots of binning errors (i.e., CRISPR and transposase genes) in MAGs. Each MAG with >90% completeness from seawater, the human gut, and the GEM was coupled with a phylogenetically close genome from RefSeq to compare MAGs and genomes from pure isolates. Seawater and human gut MAGs were coupled with GTDB genomes closest to MAGs as determined by GTDB-Tk. The MAG was excluded from this analysis if the coupled GTDB genome was unavailable in RefSeq (note that GTDB contains GenBank genomes that were ruled out from RefSeq). Regarding MAGs in GEM, counterpart genomes were determined based on the genomic operational taxonomic units defined in the GEM. The operational taxonomic units in the GEM consisted of one or more genomes from MAGs and RefSeq. For each MAG, one RefSeq genome, if any, was randomly selected from the same operational taxonomic unit. Otherwise, the MAG was not used in the analysis.

We predicted CRISPRs in each pair of MAG and its counterpart RefSeq genome using CRISPRCasFinder version 4.2.20 [47] with default parameter settings. We counted the number of CRISPR sequences with evidence levels of three or higher. We also predicted genes encoding transposases using Prodigal and KofamScan in these genome pairs. We did not compare ribosomal protein genes between the MAGs and RefSeq genomes because RefSeq excludes genomes lacking ribosomal protein genes (https://www.ncbi.nlm.nih.gov/assembly/help/anomnotrefseq/; accessed August 2, 2022).

### Bacterial growth-rate analysis

We hypothesized that tetranucleotide frequencies of ribosomal protein genes become more atypical when they are more actively translated and that their translation activities physiologically reflect bacterial growth speed. To this end, bacterial doubling time data were obtained from an integrated database of bacterial phenotypes [48]. Each entry in this database was assigned to a RefSeq genome using NCBI taxonomy IDs [49] at the species level. We used TaxonKit [50] to manage NCBI Taxonomy IDs. When multiple RefSeq genomes were available for one species, one genome was selected based on the following criteria. The reference genome was selected when available. Otherwise, a representative genome was selected for analysis. If both were unavailable, one genome was randomly chosen. When multiple pairs of genomes and data records belonged to one genus, one was randomly selected, and the others were discarded to mitigate phylogenetic biases. The CDSs of each genome were predicted and annotated using Prodigal and KofamScan, respectively. We calculated the tetranucleotide frequencies of CDSs encoding core ribosomal protein genes (Supplementary Table S1), the whole genome, and the Euclidean distances between them. The correlation between this distance and doubling time was tested using Spearman’s test in R version 4.0.5 (R Core Team, 2021).

## Results and discussion

### Unexpected absence of ribosomal protein genes from MAGs

**Fig. 1:**
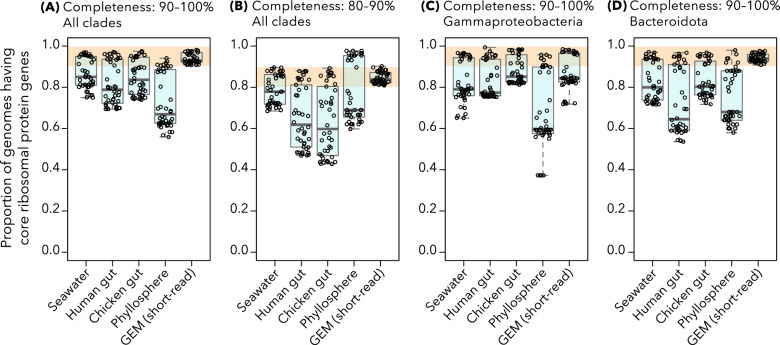
Proportion of MAGs harboring each ribosomal protein ortholog from five MAG datasets reconstructed using only short-read sequences. Each point represents one ribosomal protein ortholog (listed in Supplementary Table S1). Lines within each box and whiskers indicate three quartiles and the maximum/minimum values, respectively. Orange bands indicate the value ranges that could be expected from completeness of MAGs. Panels **A** and **B** show the results for all the bacterial MAGs with 90–100% and 80–90% completeness, respectively. Panels **C** and **D** show the results of MAGs with 90–100% completeness classified as class Gammaproteobacteria and phylum Bacteroidota, respectively. The definition of each phylum/class is slightly inconsistent between the five sets of MAGs because different versions of the GTDB were used for taxonomic annotations (Table 1).

We observed that the core ribosomal protein genes were absent in significantly more MAGs than expected by chance (e.g., MAGs with 90–100% completeness possessed less than 90% of the core ribosomal protein genes on average) in four of the five MAG datasets from different environments (Fig. 1A, B). This frequent absence of ribosomal protein genes was observed across distinct clades, and class Gammaproteobacteria and phylum Bacteroidota showed the strongest trends (Fig. 1C, D). It should be noted that CheckM (the software used for assessing genomic completeness) avoids redundant counts of adjacent ribosomal protein genes [25] and can regard genomes lacking many ribosomal protein genes as high quality.

We formulated two possible hypotheses for the unexpected absence of ribosomal protein genes in MAGs. The first hypothesis was that many environmental bacteria actually lack more ribosomal protein genes than cultivated bacteria, whose genomes are used as references [38]. The second hypothesis was that MAG assembly and binning processes could technically miss ribosomal protein genes, although they are neither repetitive nor exogenous.

**Fig. 2:**
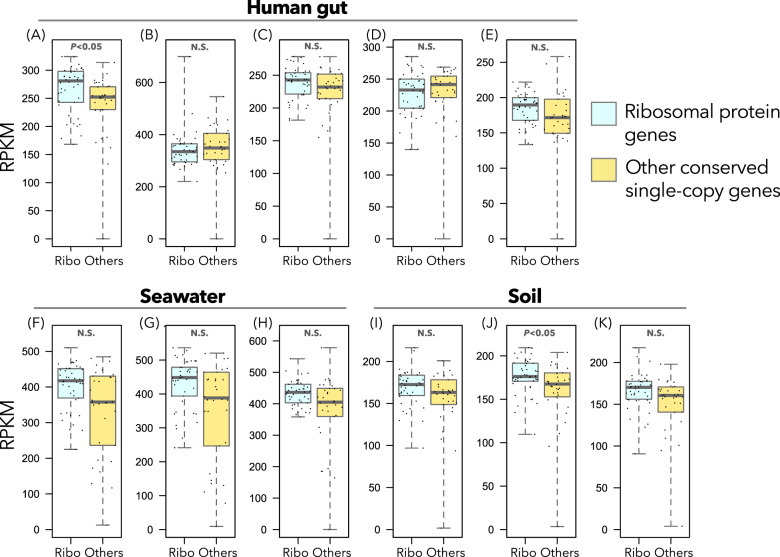
Frequencies of reads coding ribosomal protein genes and other widely conserved single-copy genes in unassembled short-read shotgun metagenomes. Each panel indicates one metagenomic sample, and each dot in a panel represents the RPKM of one ortholog (i.e., the number of reads normalized by the length of that ortholog and sequencing depth). Statistical significance (Brunner–Munzel test with Bonferroni’s correction) is shown at the top of each panel. Panels **A**–**E**, **F**–**H**, and **I**–**K** are the results from human gut metagenomes, seawater metagenomes, and soil metagenomes, respectively. N.S. not significant, Ribo ribosomal protein genes.

The first hypothesis may align with the fact that some uncultivated prokaryotes, such as Candidate Phyla Radiation bacteria, lack some ribosomal protein genes [3, 51]. However, our quantitative analysis of 11 metagenomic short-read datasets from three different environments did not support this hypothesis. The RPKMs of ribosomal protein genes (i.e., numbers of raw reads normalized by the length of each ortholog group annotated as ribosomal protein genes) were slightly higher than or not significantly different from those of the other widely conserved single-copy genes (Fig. 2: *P* < 0.05, for two samples, *P* > 0.05 for the others, with Bonferroni’s correction). This indicates that the core ribosomal protein genes exist in equimolar or slightly higher amounts, just as other single-copy genes do, and most bacterial cells harbor one copy each. Congruent with this estimation, SAGs obtained in previous studies [33, 34] showed little tendency to lack ribosomal protein genes beyond their completeness scores. All the core ribosomal protein genes were present in 89.1–97.6% of SAGs with >90% completeness. Altogether, the systematic lack of ribosomal protein genes in MAGs is likely due to a technical artifact, even if environmental Candidate Phyla Radiation and other bacteria with small ribosomes may also contribute to this result, at least partially [51].

Notably, among the five short-read MAG datasets, the GEM dataset did not show a lack of ribosomal protein genes (Fig. 1). Additionally, some bacterial clades, such as Planctomycetota, Verrucomicrobiota, and Acidobacteriota, did not exhibit this trend (Supplementary Fig. S1). Among the ribosomal protein genes, *rplM* (subunit L13, K02871), *rplS* (subunit L19, K02884), *rplT* (subunit L20, K02887), *rplU* (subunit L21, K02888), *rpmA* (subunit L27, K02899), *rpmE* (subunit L31, K02909), *rplI* (subunit L9, K02939), *rpsA* (subunit S1, K02945), *rpsO* (subunit S15, K02956), *rpsP* (subnit S16, K02959), *rpsB* (subnit S2, K02967), *rpsT* (subunit S20, K02968), *rpsF* (subunit S6, K02990), and *rpsI* (subunit S9, K02996) showed modest tendencies to be absent from the MAGs (Supplementary Fig. S2). Also, the lack of ribosomal protein genes was not observed among MAGs reconstructed using long-read sequences (Supplementary Fig. S3). While we will discuss these results later, here we would like to note that the fact that different MAG datasets show different results also supports the second hypothesis: the lack of ribosomal protein genes is likely based on technical reasons and not their intrinsic absence from environmental bacterial genomes.

### Binning errors likely cause the absence of ribosomal protein genes from MAGs

We hypothesized that the unexpected absence of ribosomal protein genes from MAGs was due to poor metagenomic assembly, binning errors, or both. We assembled the 11 shotgun metagenomic datasets (used for the raw-read analysis above) with three different sequencing depths using two renowned software packages to examine the first possibility. We obtained 66 assemblies, each consisting of 3813–93,182,511 contigs of various lengths. Soil metagenomes showed the shortest contig lengths, whereas the human gut metagenomes were assembled into longer contigs, congruent with the different alpha-diversities of these samples.

**Fig. 3:**
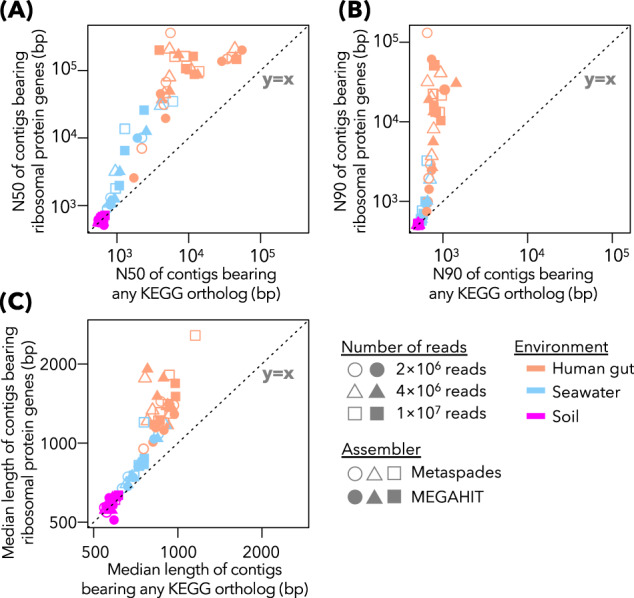
Distribution of ribosomal protein genes in assembled contigs. The relationship between N50 of all the contigs bearing any KEGG ortholog and N50 of the contigs bearing at least one ribosomal protein ortholog. Each point represents one assembly and is colored according to the source material of metagenome. The dotted line indicates y = x, where two N50 values are equal. **B** Same as **A**, although N90 was used instead of N50. **C** Same as **A**, although the median length of the contigs was used instead of N50.

We found that the N50 statistics of contigs bearing prokaryotic ribosomal protein genes were substantially larger than those of all the CDS-harboring contigs (Fig. 3A), regardless of environmental origins of samples, sequencing depths, and assemblers. This trend was consistent for the other two metrics, namely N90 and the median contig lengths (Fig. 3B, C). Therefore, a failure in metagenomic assembly is unlikely to cause the absence of ribosomal protein genes in MAGs.

**Fig. 4:**
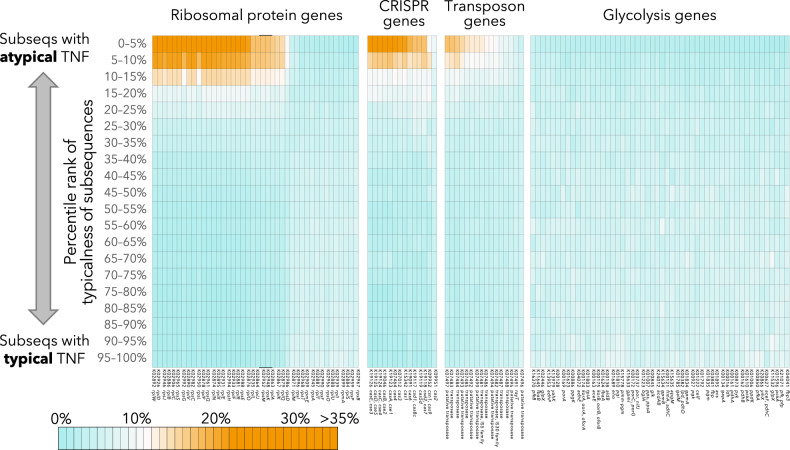
Enrichment of orthologs in subsequences with typical and atypical tetranucleotide frequencies. Each column summarizes the distribution of each ortholog among subsequences. The colors (range from blue, white, to orange) indicate the proportion of gene copies found on subsequences with specific typicalness ranks. For example, if cells in deep orange are aggregated in upper rows, that ortholog is enriched in subsequences with highly atypical tetranucleotide frequencies. TNF tetranucleotide frequency.

**Fig. 5:**
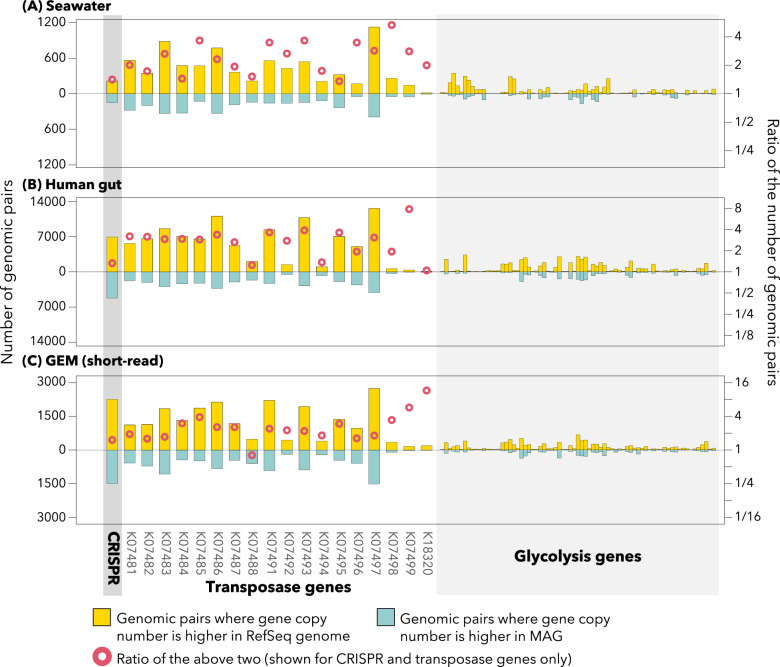
Distribution of CRISPR, transposase genes, and glycolysis genes among phylogenetically close MAGs and RefSeq genomes. The number of CRISPRs or each ortholog was counted for each MAG and phylogenetically close RefSeq genome pair. A yellow or blue bar indicates the number of pairs where the RefSeq genome or the MAG had a higher number of CRISPRs (or the ortholog), respectively (left axis). A red circle (for CRISPRs and transposase genes only) represents the ratio of these two (the former divided by the latter) (right axis, log scale). Three panels indicate the results for three different MAG datasets: seawater (**A**), the human gut (**B**), and the GEM (**C**).

Thus, we assumed that binning errors caused the absence of ribosomal protein genes in MAGs. As mentioned previously, most metagenomic binning tools harness the tetranucleotide frequencies of contigs [12] based on empirical knowledge that tetranucleotide frequencies are congruent with genomic phylogeny [18]. We split each of the 3315 RefSeq bacterial genomes that represent high-quality genomes of bacterial isolates into subsequences of 25,000–26,000 bases. Then, we calculated the tetranucleotide frequency of each subsequence to examine if tetranucleotide frequencies around ribosomal protein genes deviated from those of the whole genomic bin. As expected, most subsequences from the same genome had similar tetranucleotide frequencies. However, some presented distinct tetranucleotide frequencies, and those atypical subsequences often contained ribosomal protein genes. For example, 48.0% of the large ribosomal subunit protein gene L23 (*rplW*; K02892) were distributed in subsequences with the top 10% atypical tetranucleotide frequencies. Likewise, many ribosomal protein genes were enriched in these atypical subsequences. This enrichment level was comparable to those of exogenous sequences, such as CRISPRs and transposons (Fig. 4), which tend to be absent in MAGs with >90% completeness, compared with phylogenetically close genomes in RefSeq (i.e., genomes obtained from pure isolates), regardless of their source environments (Fig. 5). By contrast, glycolysis genes, which are non-exogenous housekeeping genes, were overall equally present in RefSeq genomes and MAGs (Fig. 5). CRISPR-related genes are often adjacent to phage-derived sequences. The genomic elements around transposons tend to be unstable and prone to duplication, deletion, or horizontal gene transfer [52–54]. Conjugal transfer genes, which originate from transferrable plasmids, were also enriched in atypical subsequences (Table 2) [55]. Thus, we concluded that atypical tetranucleotide frequencies around ribosomal protein genes led to binning errors and their absence from the MAGs. For comparison, such enrichment of atypical subsequences was not observed for glycolysis genes, which are neither exogenous nor encoded near ribosomal protein operons (Fig. 4).

**Table 2:** Ortholog groups that were enriched into subsequences of top 5% atypical tetranucleotide frequencies within each genome.

K number	Odds ratio	Definition (obtained from KEGG)
**K12063**	**10.8**	**conjugal transfer ATP-binding protein TraC**
K13012	9.45	O-antigen biosynthesis protein WbqP
**K03205**	**9.42**	**type IV secretion system protein VirD4 [EC:7.4.2.8]**
K07590	8.45	large subunit ribosomal protein L7A
K02892	8.22	large subunit ribosomal protein L23
K02926	8.20	large subunit ribosomal protein L4
K02946	8.14	small subunit ribosomal protein S10
K02886	8.02	large subunit ribosomal protein L2
K02906	8.01	large subunit ribosomal protein L3
K02965	8.00	small subunit ribosomal protein S19

Ten entries with highest odds ratio are presented. Those associated with conjugal transfer are indicated in bold letters.

We also observed that some ribosomal protein genes were not enriched in subsequences with atypical tetranucleotide frequencies (Fig. 4). Interestingly, these genes were less frequently absent from the MAGs with >90% completeness (Supplementary Fig. S2) and were sparsely distributed in the bacterial genomes, unlike the other ribosomal protein genes (Supplementary Fig. S4) [56]. In fact, genes encoded in long operons tended to be more frequently absent from MAGs compared with those encoded in small operons (Supplementary Fig. S5). In other words, ribosomal protein genes encoded in smaller operons were less prone to binning errors because of more typical contig tetranucleotide frequencies. In line with this discussion, we also note that MAGs reconstructed using long-read sequencers are less prone to the lack of ribosomal protein genes (Supplementary Fig. S3). We assume that this is because the use of long-read sequencers provides longer contigs and weakens the impact of tetranucleotide frequencies of ribosomal protein operons on those of the whole contigs.

### Bacterial life-history strategy affects the absence of ribosomal protein genes in MAGs

We then investigated why ribosomal protein genes tend to have atypical tetranucleotide frequencies, although they do not have exogenous origins. Ribosomal protein genes are characterized by low mutation rates owing to strong evolutionary constraints [57, 58]. We focused on the fact that they have unique codon usage patterns optimized for rapid translation [59, 60]. Importantly, ribosomal protein genes of fast-growing bacteria are strongly influenced by the codon usage bias compared to those of slow-growers [60].

**Fig. 6:**
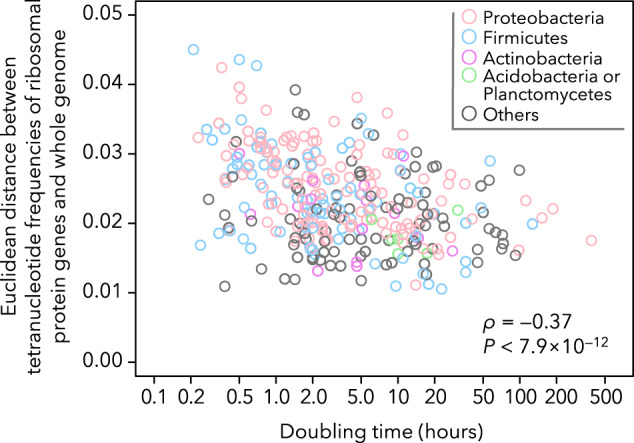
Correlation between bacterial doubling time and the degree of tetranucleotide frequency anomalies in ribosomal protein genes. The result of Spearman’s correlation test is indicated at the bottom right.

Thus, we examined whether fast-growing bacteria have more atypical tetranucleotide frequencies around ribosomal protein genes than slow-growing bacteria. We combined the genomic tetranucleotide frequency data with bacterial doubling time data. We found that Euclidean distances between tetranucleotide frequencies in the whole genome and its ribosomal protein genes significantly correlated with the doubling time of the bacterium (Spearman’s ρ = –0.37, *P* < 7.9 × 10^–12^) without any phylogenetic constraint signals (Fig. 6). In conclusion, bacterial ribosome protein genes have atypical tetranucleotide frequencies, likely because of evolutionary pressures, which are especially strong in fast-growing bacteria and lead to binning errors in the MAG reconstruction.

We can make two predictions based on this conclusion. Firstly, MAGs of bacterial groups with many slow growers should less frequently lack ribosomal protein genes. We observed that ribosomal protein genes were usually present in MAGs of Planctomycetota, Verrucomicrobiota, and Acidobacteriota, which contain many slow growers [61–63] (Supplementary Fig. S1). Consistent with this prediction, ribosomal protein genes were frequently absent from MAGs of Gammaproteobacteria and Bacteroidota (Fig. 1C, D), which are known to contain many fast growers [64, 65]. The second and related prediction is that MAGs from environmental samples that include many slow-growers and/or show low species richness (i.e., easy to conduct metagenomic binning) would lack ribosomal protein genes less frequently. We argue that the GEM dataset fulfills these two conditions because it comprises MAGs from putatively oligotrophic, low biomass, and low biodiversity samples, such as hot springs and deep subsurfaces [21].

### Conclusion and outlook

In this study, we showed that bacterial MAGs tend to lack ribosomal protein genes, in addition to well-known ribosomal RNA genes and exogenous genetic sequences. Distinct tetranucleotide frequencies around ribosomal protein genes likely cause binning errors, particularly in the genomes of fast-growing bacteria. Our conclusion cautions those who study genomics and phylogeny of uncultivated microbes, the diversity and evolution of microbial genes in the central dogma, and bioinformatics in metagenomics. For example, ribosomal protein genes form the basis of genome-based phylogeny [1].

We envision four experimental and computational solutions for this problem. First, as we saw in this study, single-cell genomic methods, which do not require a binning process, would effectively overcome this issue. However, reconstructing near-complete genomes from single cells is still difficult. Also, single-cell genomics requires costly and cumbersome cell sorting and is not readily applicable to diverse environmental samples. Second, also as we saw in this study, long-read sequencers may be used to produce long contigs. Long contigs with ribosomal protein genes contain genes with typical tetranucleotide frequencies, which enable reliable binning. Third, we may add a computational step to filter contigs with ribosomal protein genes before tetranucleotide-based binning. These contigs with ribosomal protein genes can be subsequently merged into tetranucleotide-based genomic bins by referring to the phylogenetic positions of their single-copy genes. Fourth, metagenomic binning algorithms may also be modified to give more weights on sequence depths than tetranucleotide frequencies when they deal with ribosomal protein genes.

## Supplementary information

Supplemental material

## Data Availability

All genomic and metagenomic data used in this study are publicly available in peer-reviewed literature and NCBI RefSeq, as indicated in Table 1 and Supplementary Table S2.
