# Supplementary material for: Unexpected absence of ribosomal protein genes from metagenome-assembled genomes
Source: ISME Commun. 2022 Nov 28;2:118. doi: 10.1038/s43705-022-00204-6 (PMC9723686; doi:10.1038/s43705-022-00204-6)
Supplement: Supplementary file 1 — Supplemental material [file 43705_2022_204_MOESM1_ESM.pdf]

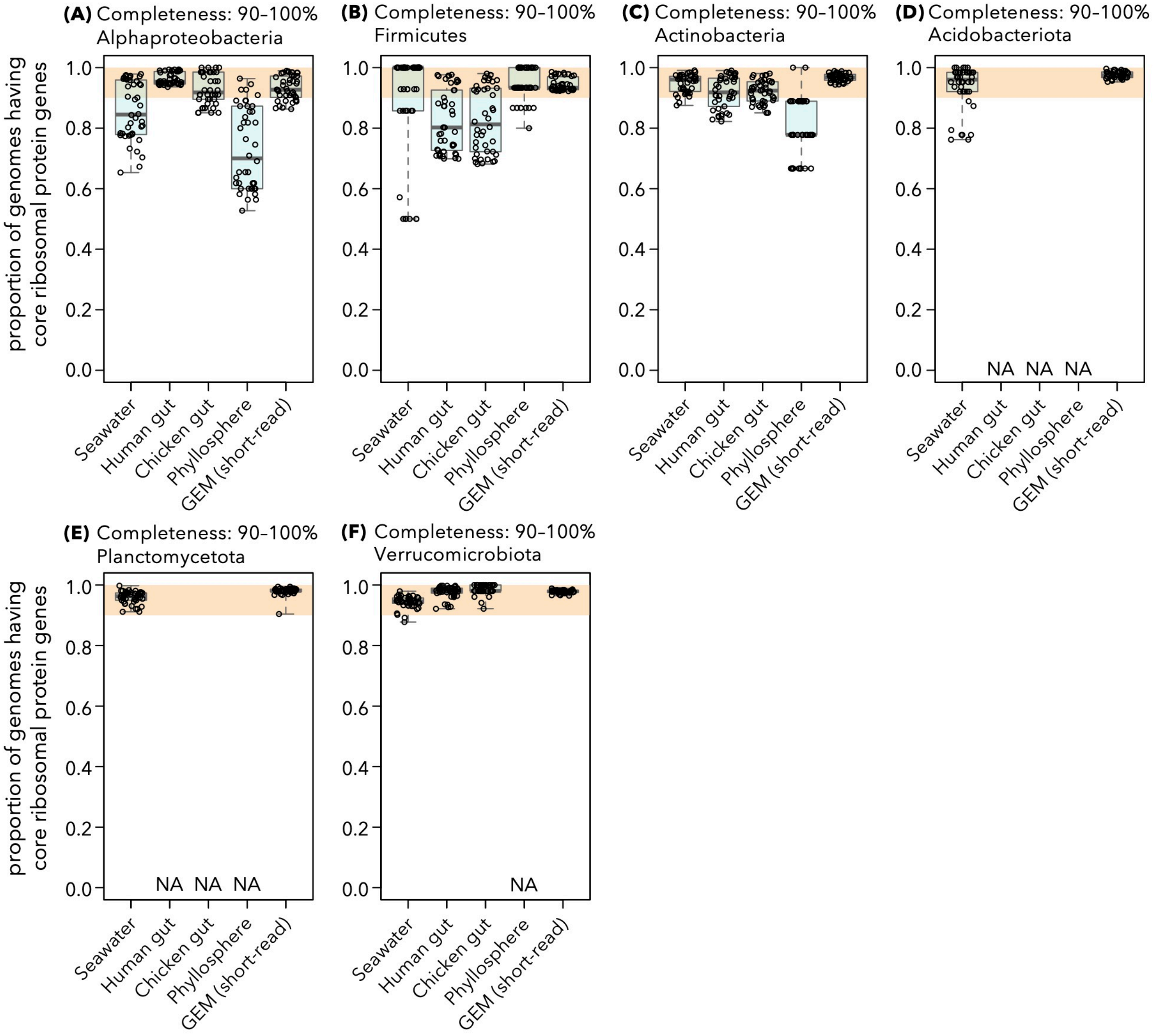

Figure S1. Proportion of MAGs harboring each ribosomal protein ortholog from five MAG datasets. Each point represents one ribosomal protein ortholog (listed in Table S1). Lines within each box and whiskers indicate three quartiles and the maximum/minimum values, respectively. Orange bands indicate the value ranges that could be expected from completeness of MAGs. The results for MAGs with 90–100% completeness that were classified as class Alphaproteobacteria (A), phylum Firmicutes (B), phylum Actinobacteriota (C), phylum Acidobacteriota (D), phylum Planctomycetota (E), or phylum Verrucomicrobiota (F) are shown. Panel (B) includes genomes assigned as any phyla starting with “Firmicutes,” such as “Firmicutes_A” and “Firmicutes_B”. NA indicates that no genomes in the dataset belonged to a specific clade. The definition of each phylum/class is slightly inconsistent between the five sets of MAGs because different versions of the GTDB were used for taxonomic annotations (Table 1).

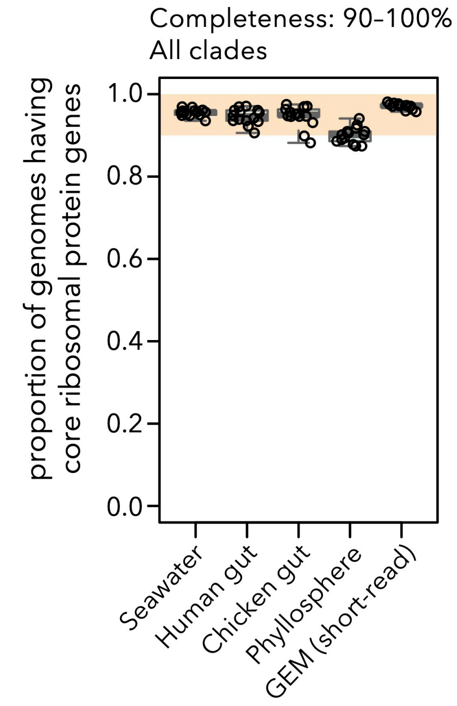

Figure S2. Proportion of MAGs harboring 14 ribosomal protein orthologs from five MAG datasets. Same as Figure 1, but only 14 ribosomal protein orthologs, namely *rplM* (K02871), *rplS* (K02884), *rplT* (K02887), *rplU* (K02888), *rpmA* (K02899), *rpmE* (K02909), *rplI* (K02939), *rpsA* (K02945), *rpsO* (K02956), *rpsP* (K02959), *rpsB* (K02967), *rpsT* (K02968), *rpsF* (K02990), and *rpsI* (K02996) in KEGG, are indicated. Each point represents one of these orthologs. Lines within each box and whiskers indicate three quartiles and the maximum/minimum values, respectively. Orange band indicates the value ranges that could be expected from completeness of MAGs (i.e., 90–100%). Only bacterial MAGs with 90–100% completeness were used.

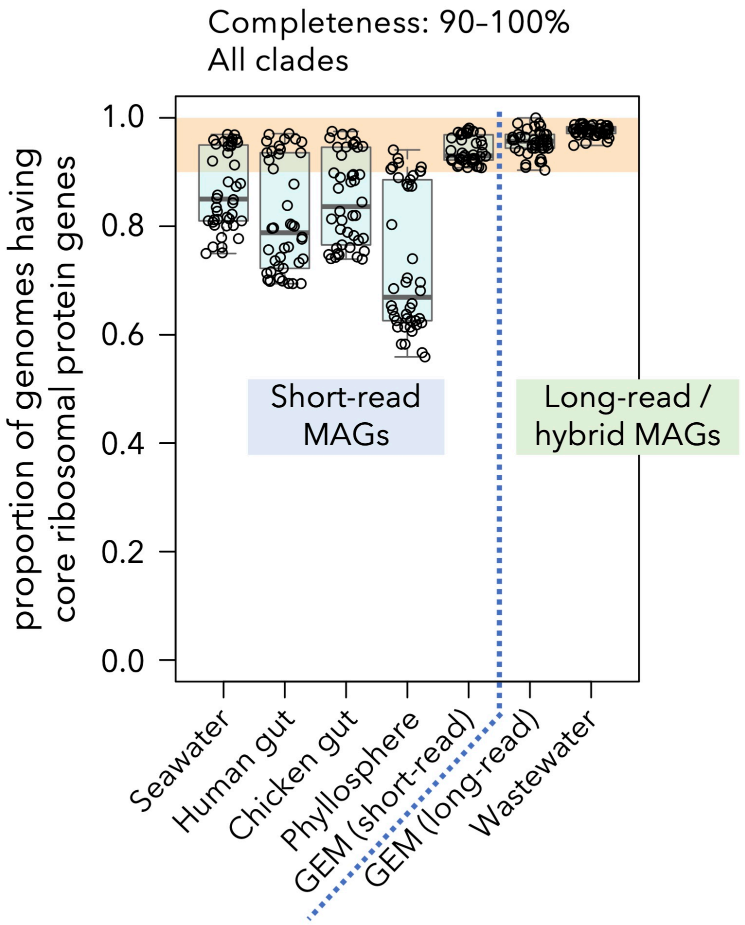

Figure S3. Frequency of each ribosomal protein gene on MAGs constructed with and without long-read sequences. Same as Figure 1, but two additional MAG datasets that have been constructed using long-read sequencers have been added. Only bacterial MAGs with 90–100% completeness were used. Each point represents one ribosomal protein ortholog (listed in Table S1). Lines within each box and whiskers indicate three quartiles and the maximum/minimum values, respectively. Orange band indicates the value ranges that could be expected from completeness of MAGs (i.e., 90–100%). Five columns on the left indicate the results for MAGs constructed without long-read sequences, whereas the two columns on the right indicate ones for MAGs constructed using long-read sequences.

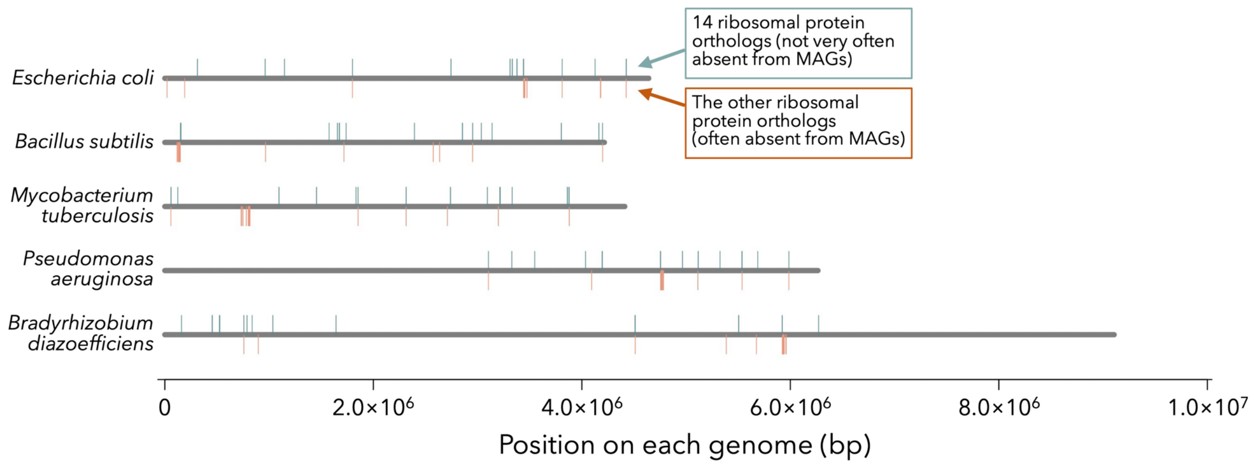

Figure S4. Distributions of ribosomal protein genes in bacterial genomes. Each horizontal line indicates one genome (all five consist of only one contig each), and vertical lines denote the positions of ribosomal protein genes. Blue lines indicate the positions of 14 ribosomal protein orthologs (see Figure S2), which were infrequently absent from MAGs. Orange lines indicate the positions of the other ribosomal proteins.

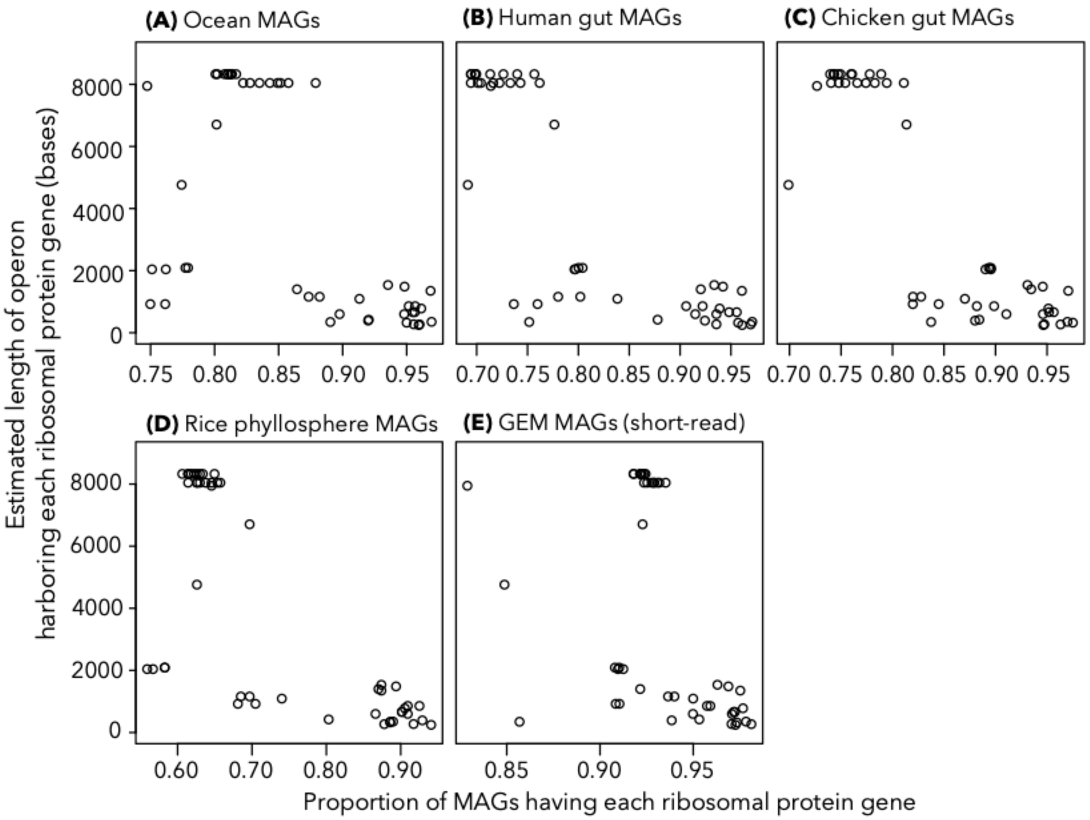

Figure S5. Correlation between the proportion of MAGs harboring ribosomal protein gene and the length of operon containing that ribosomal protein gene. Each point represents one ribosomal protein ortholog (listed in Table S1). Panels (A)–(E) indicate the results for seawater MAGs, human gut MAGs, chicken gut MAGs, rice phyllosphere MAGs, and GEM MAGs (only short-read ones), respectively.

Table S1. KEGG ortholog numbers determined as core ribosomal protein genes and single-copy genes that are conserved in most prokaryotic genomes.

Category	K numbers	Gene name / description	
Ribosomal protein genes	K02863	*rplA*	large subunit ribosomal protein L1
	K02864	*rplJ*	large subunit ribosomal protein L10
	K02867	*rplK*	large subunit ribosomal protein L11
	K02871	*rplM*	large subunit ribosomal protein L13
	K02874	*rplN*	large subunit ribosomal protein L14
	K02876	*rplO*	large subunit ribosomal protein L15
	K02878	*rplP*	large subunit ribosomal protein L16
	K02879	*rplQ*	large subunit ribosomal protein L17
	K02881	*rplR*	large subunit ribosomal protein L18
	K02884	*rplS*	large subunit ribosomal protein L19
	K02886	*rplB*	large subunit ribosomal protein L2
	K02887	*rplT*	large subunit ribosomal protein L20
	K02888	*rplU*	large subunit ribosomal protein L21
	K02890	*rplV*	large subunit ribosomal protein L22
	K02892	*rplW*	large subunit ribosomal protein L23
	K02895	*rplX*	large subunit ribosomal protein L24
	K02899	*rpmA*	large subunit ribosomal protein L27
	K02906	*rplC*	large subunit ribosomal protein L3
	K02909	*rpmE*	large subunit ribosomal protein L31
	K02926	*rplD*	large subunit ribosomal protein L4
	K02931	*rplE*	large subunit ribosomal protein L5
	K02933	*rplF*	large subunit ribosomal protein L6
	K02935	*rplL*	large subunit ribosomal protein L7/L12
	K02939	*rplI*	large subunit ribosomal protein L9
	K02945	*rpsA*	small subunit ribosomal protein S1
	K02946	*rpsJ*	small subunit ribosomal protein S10
	K02948	*rpsK*	small subunit ribosomal protein S11
	K02950	*rpsL*	small subunit ribosomal protein S12
	K02952	*rpsM*	small subunit ribosomal protein S13
	K02956	*rpsO*	small subunit ribosomal protein S15
	K02959	*rpsP*	small subunit ribosomal protein S16
	K02961	*rpsQ*	small subunit ribosomal protein S17
	K02965	*rpsS*	small subunit ribosomal protein S19
	K02967	*rpsB*	small subunit ribosomal protein S2
	K02968	*rpsT*	small subunit ribosomal protein S20
	K02982	*rpsC*	small subunit ribosomal protein S3
	K02986	*rpsD*	small subunit ribosomal protein S4
	K02988	*rpsE*	small subunit ribosomal protein S5
	K02990	*rpsF*	small subunit ribosomal protein S6
	K02992	*rpsG*	small subunit ribosomal protein S7
	K02994	*rpsH*	small subunit ribosomal protein S8
	K02996	*rpsI*	small subunit ribosomal protein S9

Table S1: continued

Category	K numbers	Gene name / description	
Conserved single-copy genes	K00554	*trmD*	tRNA (guanine37-N1)-methyltransferase [EC:2.1.1.228]
	K00962	*pnp*	polyribonucleotide nucleotidyltransferase [EC:2.7.7.8]
	K01409		N6-L-threonylcarbamoyladenine synthase [EC:2.3.1.234]
	K01869	*leuS*	leucyl-tRNA synthetase [EC:6.1.1.4]
	K01870	*ileS*	isoleucyl-tRNA synthetase [EC:6.1.1.5]
	K01875	*serS*	seryl-tRNA synthetase [EC:6.1.1.11]
	K01887	*argS*	arginyl-tRNA synthetase [EC:6.1.1.19]
	K01889	*pheS*	phenylalanyl-tRNA synthetase alpha chain [EC:6.1.1.20]
	K01890	*pheT*	phenylalanyl-tRNA synthetase beta chain [EC:6.1.1.20]
	K01892	*hisS*	histidyl-tRNA synthetase [EC:6.1.1.21]
	K02343	*dnaX*	DNA polymerase III subunit gamma/tau [EC:2.7.7.7]
	K02520	*infC*	translation initiation factor IF-3
	K02600	*nusA*	transcription termination/antitermination protein NusA
	K02601	*nusG*	transcription termination/antitermination protein NusG
	K02835	*prfA*	peptide chain release factor 1
	K02836	*prfB*	peptide chain release factor 2
	K02838	*frr*	ribosome recycling factor
	K03046	*rpoC*	DNA-directed RNA polymerase subunit beta' [EC:2.7.7.6]
	K03076	*secY*	preprotein translocase subunit SecY
	K03177	*truB*	tRNA pseudouridine55 synthase [EC:5.4.99.25]
	K03545	*tig*	trigger factor
	K03550	*ruvA*	holliday junction DNA helicase RuvA [EC:5.6.2.4]
	K03551	*ruvB*	holliday junction DNA helicase RuvB [EC:5.6.2.4]
	K03553	*recA*	recombination protein RecA
	K03596	*lepA*	GTP-binding protein LepA
	K03625	*nusB*	transcription antitermination protein NusB
	K03664	*smpB*	SsrA-binding protein
	K03702	*uvrB*	excinuclease ABC subunit B
	K03977	*engA*	GTPase
	K03979	*obgE*	GTPase [EC:3.6.5.-]
	K04075	*tilS*	tRNA(Ile)-lysidine synthase [EC:6.3.4.19]
	K06187	*recR*	recombination protein RecR

Table S2. Unassembled shotgun metagenomic datasets used for this study.

Source Environment	Reference	INSDC accession number
Human gut	Hildebrand et al., 2021	ERR4794107
		ERR4794313
		ERR4794328
		ERR4794333
		ERR4794596
Seawater	Sunagawa et al., 2015	ERR598950
		ERR599066
		ERR599090
Soil	Li et al., 2018	SRR5512140
		SRR5512146
		SRR5512147

Table S3. A list of 103 tetranucleotides that appears in mutually independent frequency.

CATT	CCGT	CCTT	CGCT	CGGT	CGTG	CGTT	CTAT	CTCT	CTGG	CTGT	CTTG
CTTT	GAAT	GACT	GAGT	GATC	GATG	GATT	GCAT	GCCT	GCGG	GCGT	GCTC
GCTG	GCTT	GGAT	GGCC	GGCG	GGCT	GGGC	GGGG	GGGT	GGTC	GGTG	GGTT
GTAG	GTAT	GTCC	GTCG	GTCT	GTGC	GTGG	GTGT	GTTC	GTTG	GTTT	TAAG
TAAT	TACC	TACG	TACT	TAGC	TAGG	TAGT	TATA	TATC	TATG	TATT	TCAG
TCAT	TCCC	TCCG	TCCT	TCGA	TCGC	TCGG	TCGT	TCTA	TCTC	TCTG	TCTT
TGAC	TGAG	TGAT	TGCA	TGCC	TGCG	TGCT	TGGA	TGGC	TGGG	TGGT	TGTA
TGTC	TGTG	TGTT	TTAA	TTAC	TTAG	TTAT	TTCA	TTCC	TTCG	TTCT	TTGA
TTGC	TTGG	TTGT	TTTA	TTTC	TTTG	TTTT					
